# Human variome project – current overview

**DOI:** 10.1186/1755-8166-7-S1-I1

**Published:** 2014-01-21

**Authors:** Richard Cotton

**Affiliations:** 1 Human Variome Project International Limited, Melbourne, Australia; 2 Department of Pathology, The University of Melbourne, Australia

## 

The Human Variome Project (HVP) was initiated in 2006 to foster discussion around how patient outcomes could be improved by connecting and promoting the disparate work of genetic variation database curators. In the six years that have passed since that meeting, the concept of the Human Variome Project has evolved and matured. The challenges and complexities that face the field of human genetics, form a constantly changing technology landscape. The influx of raw data that those changes bring, has necessitated a shift away from the Project’s initial passive stance—a forum for the sharing of ideas and collaboration—to a more active initiative that actively engages with partners and stakeholders to establish and maintain the standards, systems and infrastructure that will enable the global collection and sharing of genetic variation information to be integrated into routine clinical practice. The Project is now well established, with over 900 HVP consortium members from 72 countries, 16 countries officially developing HVP country nodes, 6 major disease groups developing databases and over 140 databases having joined the Gene/Disease Specific Advisory Council.

With the *Project Roadmap 2010-2012* nearing completion, the establishment of a new committee structure to govern the scientific aspects of the Project’s activities and the incorporation of a legal entity, Human Variome Project International Limited, to act as the Project’s International Coordinating Office outlined in the Roadmap are now in place. Most importantly, the Pro*ject Roadmap 2010-2012* proposed a strategy that would ultimately ensure the collection of all genetic variation information worldwide—the *One Project*, *Two Channels*, *Multiple Locations Strategy*. This strategy, the next *Project Roadmap 2012-2016* and progress will be outlined in the presentation.

